# Triglyceride-glucose index is significantly associated with the risk of hyperuricemia in patients with diabetic kidney disease

**DOI:** 10.1038/s41598-022-23478-1

**Published:** 2022-11-21

**Authors:** Qiuhong Li, Xian Shao, Saijun Zhou, Zhuang Cui, Hongyan Liu, Tongdan Wang, Xiaoguang Fan, Pei Yu

**Affiliations:** 1 NHC Key Laboratory of Hormones and Development, Chu Hsien-I Memorial Hospital and Tianjin Institute of Endocrinology, Tianjin Medical University, Tianjin, 300134 China; 2 Tianjin Key Laboratory of Metabolic Diseases, Tianjin Medical University, Tianjin, 300134 China; 3 Department of Epidemiology and Health Statistics, Tianjin Medical University, Tianjin, 300010 China; 4 Department of Nephrology, Henan Provincial People’s Hospital, Department of Nephrology of Central China Fuwai Hospital, Central China Fuwai Hospital of Zhengzhou University, Zhengzhou Henan, 450003 China

**Keywords:** Biomarkers, Endocrinology

## Abstract

Triglyceride–glucose (TyG) index has been proposed to be a simple, economical, and reliable marker of insulin resistance. We aimed to investigate whether TyG is an independent predictor of hyperuricemia in diabetic kidney disease (DKD) populations by conducting a cross-sectional and longitudinal study. A total of 6,471 patients were enrolled in cross-sectional analysis, and 3,634 patients without hyperuricemia at the baseline were included in longitudinal analysis and were followed up for a median of 23.0 months. Hyperuricemia was categorized as a serum uric acid level ≥ 420 umol/L (7 mg/dL). In this study, 19.58% of participants had hyperuricemia. In the cross-sectional analysis, multivariate logistics regression analysis showed that the ORs (95% CI) for hyperuricemia in the second, third, and fourth TyG quartiles were 1.40 (95% CI 0.73–2.65), 1.69 (95% CI 0.90–3.18), and 4.53 (95% CI 2.39–8.57), respectively, compared with the first quartile. Longitudinally, the Kaplan–Meier survival analysis showed that higher TyG levels predicted higher incidence of hyperuricemia. Multivariate Cox regression model revealed that the hazard ratios for hyperuricemia in the upper quartiles of the TyG index were 1.69 (95% CI 0.97–2.93), 2.23 (95% CI 1.33–3.75), and 2.50 (95% CI 1.46–4.27), respectively, compared with the first quartile. Moreover, the subgroup analyses revealed that the relationship between TyG levels and hyperuricemia was robust in DKD patients. Our findings indicate a significant independent correlation between the TyG index and the risk of hyperuricemia in DKD patients.

## Introduction

Hyperuricemia has become a global health problem due to an improving standard of living and rapid economic growth^1,2^. Recent epidemiological investigations have estimated the incidence of hyperuricemia in adults is approximately 21% in the United States^3^, 13.3% in mainland China^4^, 20% in the urban areas of Mexico^5^, and 25.8% in Japan^6^. It has been shown that hyperuricemia not only plays an important role in the development of gout, which can impair patient quality of life^7^, but is also an independent risk factor for many diseases, including hypertension^8^, type 2 diabetes^9^, dyslipidemia^10^, stroke^11^, chronic kidney disease^12^, and cardiovascular events^13^, which increase the risk of morbidity and mortality. However, due to the complexity of metabolic regulation, the pathogenesis of hyperuricemia remains unclear. Therefore, early identification of high-risk groups of hyperuricemia can enable them to receive early intervention, which is very meaningful for improving patient quality of life and alleviate the burden placed on the healthcare system and the economy.

The triglyceride-glucose (TyG) index is combination of fasting plasma glucose and triglycerides^14^, and can be used as a simple, economical, and reliable substitute maker of insulin resistance, compared with HOMA-IR^15,16^. Studies have shown that insulin resistance is a pathophysiological process that is closely associated with hyperuricemia^17^,and hyperinsulinemia caused by insulin resistance can cause renal urate excretion reduce, resulting in blood urate accumulation^18^. Recent studies have revealed the correlation between TyG index and hyperuricemia, and the correlation was stronger than that between obesity indices and hyperuricemia among general Chinese populations^19–22^. In addition, in a cross-sectional study, the TyG index and hyperuricemia were also positively correlated in hypertensive adults^23^.

Research has shown that patients with diabetes are more insulin resistant (IR) in the context of renal disease^24^. However, in patients with diabetic kidney disease (DKD), studies on the relationship between the TyG index and hyperuricemia are limited. Thus, the aim of our study was to explore the relationship between the TyG and the risk of hyperuricemia, after adjusting for known influencing factors, to evaluate the value of TyG for optimizing the risk stratification and prevention of hyperuricemia in the DKD population.

## Results

### Baseline characteristics

**Figure 1:**
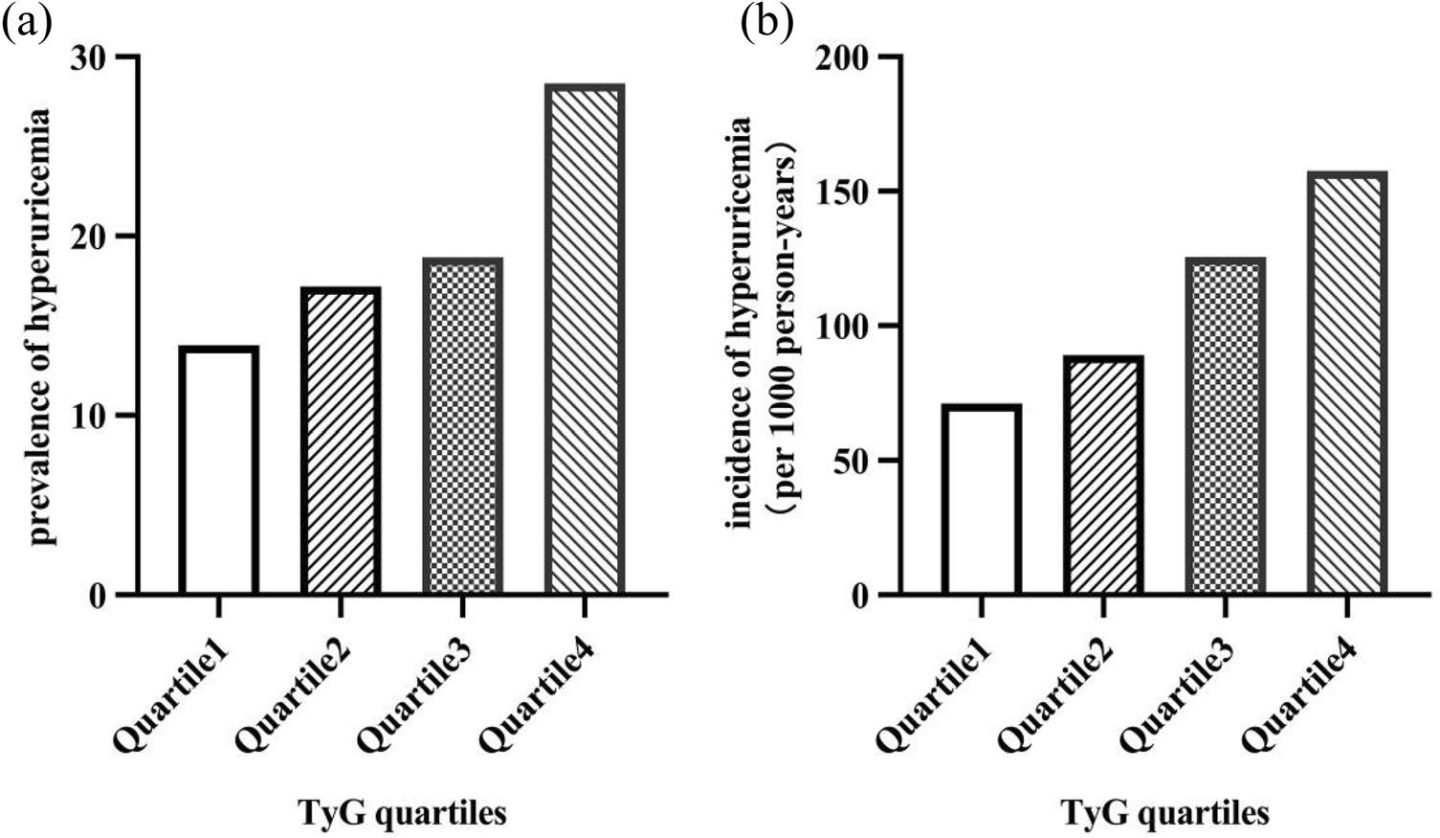
Relationship between baseline TyG and the hyperuricemia (**a**) the prevalence of hyperuricemia in the cross-sectional analysis, by baseline TyG quartile: < 9.02; quartile 2, 9.02–9.45 quartile 3, 9.45–9.96; quartile 4, ≥ 9.96; (**b**) the incidence of hyperuricemia ( per 1,000 person-years) in the cohort analysis, by baseline TyG quartile: quartile 1, < 9.00; quartile 2, 9.00–9.42; quartile 3, 9.42–9.90; quartile 4, ≥ 9.90;

A total of 6,471 participants were enrolled in the cross-sectional study. The clinical characteristics of the participants were stratified using TyG quartiles, as shown in Table 1. The mean age at the baseline was 59.11 ± 10.53 years, and included 3,780 men (58.41%; age, 57.45 ± 10.99 years) and 2,691 women (41.59%; age, 61.44 ± 9.36 years). The prevalence of hyperuricemia was 19.58% (males, 23.99%; females, 13.38%). The prevalence of hyperuricemia was significantly increased with increasing TyG quartile (13.9%, 17.2%, 18.8%, and 28.5%, respectively, for the first, second, third, and fourth quartiles; *P* < 0.05 for the trend) (Fig. 1a). In comparison with patients with lower TyG levels, patients with higher TyG levels had higher DBP, BMI, HbA1c, TC, LDL-C, BUN, eGFR, Blood uric acid, 24hTP and ALT levels but lower levels of Bilirubin, HDL-C, and LDH (*P* < 0.05 for each).

**Table 1:** Baseline characteristics of the subjects stratified using TyG quartiles.

Characteristic	TyG quartiles				*P* value
	Quartile 1	Quartile 2	Quartile 3	Quartile 4	
N	1604	1625	1636	1606	
Age (y)	61.38 ± 9.59	60.36 ± 9.57	59.32 ± 10.38	55.36 ± 11.48	0.000
Male, n (%)	990 (61.7)	893 (55)	922 (56.4)	975 (60.7)	0.000
Hypertension, n (%)	284 (76.1)	363 (77.4)	393 (73.5)	487 (76.6)	0.478
SBP (mmHg)	138.08 ± 24.33	140.07 ± 23.32	141.91 ± 20.52	140.43 ± 22.75	0.055
DBP (mmHg)	79.69 ± 13.30	81.42 ± 12.85	82.77 ± 12.31	83.53 ± 13.15	0.000
BMI (kg/m^2^)	26.41 ± 3.75	26.96 ± 3.86	27.60 ± 3.87	28.07 ± 4.11	0.000
FPG (mmol/L)	7.54 ± 1.80	9.02 ± 2.35	10.43 ± 2.92	13.05 ± 4.10	0.000
HbA1c (%)	7.45 ± 1.48	7.97 ± 1.60	8.51 ± 1.75	9.39 ± 1.87	0.000
TG (mmol/L)	0.99 (0.80–1.20)	1.48 (1.25–1.75)	2.02 (1.67–2.45)	3.51 (2.62–5.16)	0.000
TC (mmol/L)	4.73 ± 1.10	5.22 ± 1.17	5.49 ± 1.23	6.08 ± 1.79	0.000
LDL-C (mmol/L)	3.02 (2.43–3.60)	3.39 (2.80–4.00)	3.53 (2.86–4.16)	3.43 (2.81–4.25)	0.000
HDL-C (mmol/L)	1.37 ± 0.34	1.29 ± 0.28	1.23 ± 0.26	1.18 ± 0.24	0.000
BUN (mmol/L)	5.80 (4.84–7.14)	5.80 (4.82–7.13)	5.82 (4.77–7.12)	6.01 (4.93–7.43)	0.002
SCR (umol/L)	70.30 (57.50–86.68)	69.10 (57.15–85.10)	69.10 (57.00–86.18)	69.70 (57.80–88.40)	0.574
eGFR (mL/min/1.73 m^2^)	95.18 ± 30.22	95.20 ± 30.73	95.66 ± 31.68	98.36 ± 34.33	0.018
SUA (umol/L)	325.75 ± 91.31	337.82 ± 96.87	344.46 ± 97.55	374.36 ± 104.63	0.000
Hyperuricemia, n (%)	223 (13.9)	279 (17.2)	308 (18.8)	457 (28.5)	0.000
TBIL (umol/L)	12.50 (9.50–16.10)	12.50 (9.80–16.50)	12.30 (9.60–15.80)	12.00 (9.15–15.20)	0.001
DBIL (umol/L)	3.80 (2.70–5.30)	3.50 (2.60–4.90)	3.30 (2.38–4.60)	3.00 (2.10–4.10)	0.000
AST (U/L)	18.60 (15.60–22.60)	18.90 (15.90–23.30)	18.60 (15.50–23.80)	18.60 (15.10–24.20)	0.326
ALT (U/L)	18.20 (13.10–25.05)	19.60 (14.20–27.60)	20.30 (14.50–29.45)	21.10 (14.60–31.95)	0.000
LDH (U/L)	187.11 ± 47.88	181.56 ± 36.90	182.63 ± 41.85	175.98 ± 36.57	0.001
ACR (mg/g)	99.56(28.19–415.34)	77.05(29.93–439.65)	117.26(26.53–408.94)	169.05 (51.80–464.05)	0.007
24hTP (g/d)	0.23(0.13–0.92)	0.24(0.13–0.96)	0.28(0.13–1.15)	0.35(0.15–1.50)	0.000
TyG	8.65 ± 0.29	9.24 ± 0.12	9.69 ± 0.15	10.56 ± 0.57	0.000

*TyG* triglyceride-glucose index; *SBP* systolic blood pressure; *DBP* diastolic blood pressure; *BMI* body mass index; *FPG* fasting plasma glucose; *HbA1c* glycated hemoglobin; *TG* plasma triglyceride level; *TC* total cholesterol; *LDL-C* low-density lipoprotein cholesterol; *HDL-C* high-density lipoprotein cholesterol; *BUN* blood urea nitrogen; *SCR* serum creatinine; *eGFR* estimated glomerular filtration rate; *SUA* serum uric acid; *TBIL* total bilirubin; *DBIL* direct bilirubin; *AST* aspartate aminotransferase; *ALT* alanine aminotransferase; *LDH* lactic dehydrogenase; *ACR* albumin-creatinine ratio; *24hTP* 24 h total urine protein;

Data are presented as the mean ± standard error or median (interquartile range) for continuous variables, and the numbers (percentage) for categorical variables.

*P* < 0.05 was considered statistically significant.

### The associations of the TyG index and hyperuricemia

We conducted multivariate logistic regression to show the odds ratios (ORs) and 95% CI for hyperuricemia based on the TyG index quartiles (Table 2). In unadjusted model (model 1), the ORs (95% CI) for hyperuricemia in the second, third, and fourth TyG quartiles were 1.28 (95% CI 1.06–1.55), 1.44 (95% CI 1.19–1.73), and 2.46 (95% CI 2.06–2.94), respectively, compared with the first quartile. After adjustment for age and sex (model 2), the ORs (95% CI) were 1.33 (95% CI 1.10–1.62), 1.47 (95% CI 1.21–1.78), and 2.40 (95% CI 1.99–2.88), respectively. After adjusting for age, sex, HDL-C, LDL-C, BMI, eGFR, SBP, DBP, 24hTP, HbA1c (model 3), the relationship was still significant, with ORs (95% CIs) of 1.40 (95% CI 0.73–2.65), 1.69 (95% CI 0.90–3.18), and 4.53 (95% CI 2.39–8.57), respectively. Our findings revealed that the TyG index and hyperuricemia were independently correlated in DKD patients.

**Table 2:** Cross-sectional analysis of the association between the baseline TyG index and the incidence of hyperuricemia.

Variables	Odds ratio (95% CI)					
	Model1	*P* value	Model 2	*P* value	Model 3	*P* value
Quartiles of TyG		0.000		0.000		0.000
Quartile 1	Reference		Reference		Reference	
Quartile 2	1.28 (1.06–1.55)	0.011	1.33 (1.10–1.62)	0.003	1.40 (0.73–2.65)	0.310
Quartile 3	1.44 (1.19–1.73)	0.000	1.47 (1.21–1.78)	0.000	1.69 (0.90–3.18)	0.105
Quartile 4	2.46 (2.06–2.94)	0.000	2.40 (1.99–2.88)	0.000	4.53 (2.39–8.57)	0.000

*CI* confidence interval.

Model 1: no adjustment; Model 2: adjusted for age, gender.

Model 3: adjusted for all the factors in model 1 and HDL-C, LDL-C, BMI, eGFR, 24hTP, SBP, DBP, HbA1c.

### The associations of the TyG index and the incidence of hyperuricemia

During the median follow-up of 23.0 months (IQR 8.0, 49.25), 970 of the 3,634 participants developed hyperuricemia. We revealed that an increased TyG index was associated with an increased risk of hyperuricemia. The incidence of the hyperuricemia was 71.08 per 1000 person-years (20.0%), 89.15 per 1000 person-years (23.4%), 125.53 per 1000 person-years (28.9%), 157.63 per 1000 person-years (34.2%), respectively, for the first, second, third, and fourth quartiles. (*P* < 0.05 for the trend) (Fig. 1b).

**Figure 2:**
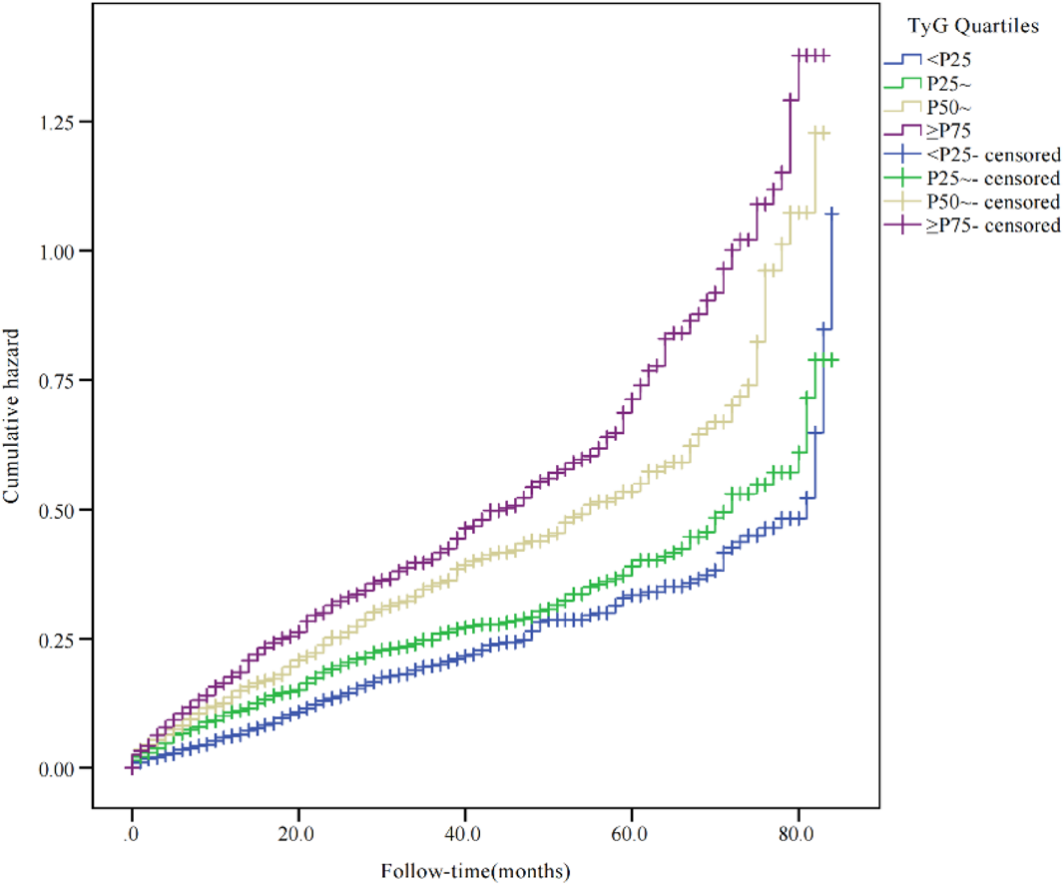
Kaplan–Meier curves for hyperuricemia by TyG quartile: quartile 1 (< 25), < 9.00; quartile 2 (25 ~), 9.00–9.42; quartile 3 (50 ~), 9.42–9.90; and quartile 4 (≥ 75), ≥ 9.90. *P* value < 0.05 for log-rank test, other quartiles versus first quartile.

The Kaplan–Meier survival analysis (Fig. 2) revealed that the risk of hyperuricemia was based on TyG quartiles category (*P* for log-rank test < 0.001). The lowest quartile of the 4 groups indicated the lowest level disease hazard for hyperuricemia, while the highest quartile indicated the highest level of disease hazard.

We further used the multivariate Cox regression model to test the associations of the TyG index and the incidence of hyperuricemia in all models (Table 3). After full adjustment based on age, sex, LDL-C, HDL-C, BMI, eGFR, 24hTP, SBP, DBP, and HbA1c (model 3), the hazard ratios for hyperuricemia in the upper quartiles of the TyG index were 1.69 (95% CI 0.97–2.93), 2.23 (95% CI 1.33–3.75), and 2.50 (95% CI 1.46–4.27), respectively, compared with the first quartile.

**Table 3:** Cohort analysis of the association between the baseline TyG index and the incidence of hyperuricemia.

Variables	Hazard ratio (95% CI)					
	Model 1	*P* value	Model 2	*P* value	Model 3	*P* value
Quartiles of TyG		0.000		0.000		0.006
Quartile 1	Reference		Reference		Reference	
Quartile 2	1.25 (1.03–1.53)	0.028	1.29 (1.05–1.57)	0.014	1.69 (0.97–2.93)	0.062
Quartile 3	1.80 (1.48–2.18)	0.000	1.82 (1.50–2.21)	0.000	2.23 (1.33–3.75)	0.002
Quartile 4	2.27 (1.88–2.73)	0.000	2.32 (1.92–2.80)	0.000	2.50 (1.46–4.27)	0.001

*CI* confidence interval.

Model 1: no adjustment; Model 2: adjusted for age, gender.

Model 3: adjusted for all the factors in model 1 and HDL-C, LDL-C, BMI, eGFR, 24hTP, SBP, DBP, HbA1c.

### Subgroup analyses

**Figure 3:**
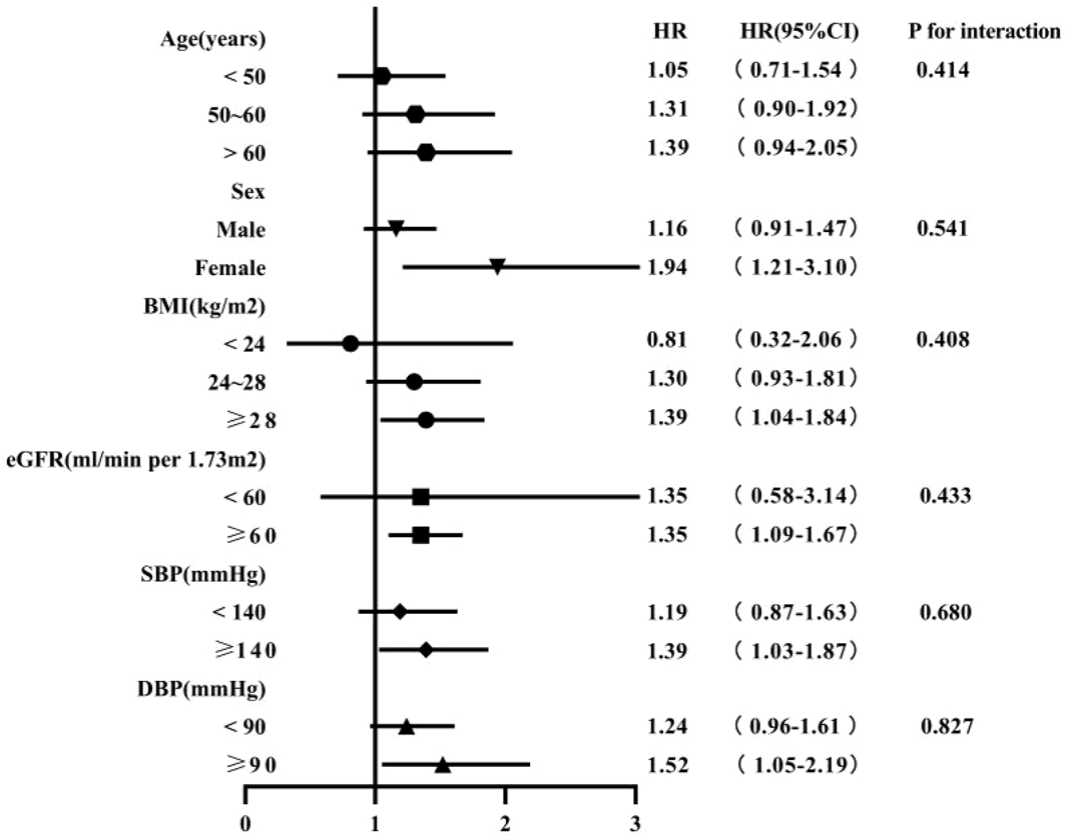
Stratified analyses of the association between the TyG index and the incidence of hyperuricemia. Adjusted for age, sex,HDL-C, LDL-C, BMI, eGFR, 24hTP, SBP, DBP and HbA1c.

We further assessed the relationships of TyG index and the incidence of hyperuricemia stratified by age, sex, BMI, eGFR, SBP, and DBP (Fig. 3). No significant heterogeneity was found in the following stratified analysis, including age (< 50 vs. 50 ~ 60 vs. > 60 years), sex (Male vs. Female), BMI (< 24 vs.24 ~ 28 ≥ 28 kg/m^2^), eGFR (< 60 vs. ≥ 60 mL/min/1.73 m^2^), SBP (< 140 vs. ≥ 140 mmHg), and DBP (< 90 vs. ≥ 90 mmHg) (*P* values > 0.05 for all). The association between the TyG index and the incidence of hyperuricemia was consistent within all subgroups.

## Discussion

As far as we know, this is the first cross-sectional and longitudinal study to examine the relationship between TyG index and hyperuricemia in DKD populations. After adjusting for potential confounding factors of hyperuricemia, our study revealed that the TyG index could independently predict the incidence of hyperuricemia. In addition, our findings were robust across major demographic and clinical subgroups.

In recent years, researchers have explored the relationships between the TyG index and hyperuricemia in the general and hypertensive populations. Shi et al.^21^ performed cross-sectional studies on 6,466 subjects and revealed that TyG was positively related to hyperuricemia after adjusting for potential confounding factors. Kahaer et al.^22^ performed a cross-sectional analysis on 2,243 patients in a medical check-up population from Xinjiang, China, and the results revealed that the TyG index was more closely associate with hyperuricemia than obesity indices. Yu et al.^23^ performed a cross-sectional study on adults with hypertension, and showed that a higher level of TyG was associated with a higher risk of hyperuricemia. A cohort study conducted by Qing et al.^19^ on a general Chinese population found an increase in the risk of hyperuricemia for the upper TyG quartiles, after multivariate adjustment. Consistent with the results of the above-mentioned studies, our findings further revealed that TyG index levels are stable and independently related with the incidence of hyperuricemia in the DKD population through cross-sectional and longitudinal analysis. Taken together, these results suggest that the TyG index can predict hyperuricemia in a larger population as well.

Epidemiologically, it is widely known that the prevalence of hyperuricemia varies between different populations and geological locations, and may even vary a lot between different provinces^25^. According to our study, 19.58% of the participants had hyperuricemia, which is similar to that reported in T2DM populations in Tianjin (17.24%)^26^, Shanghai (18.7%)^27^ and in patients with early stage DKD (20.69%)^28^. Our study population was DKD patients not only accompanied by insulin resistance but also hyperinsulinemia due to insulin resistance and also included patients with impaired kidney function. Studies have shown that uric acid levels are dependent on purine production, excretion, and absorption^29^, and that 70% of daily UA production is eliminated by the kidney^30^. Thus, due to the effects of kidney injury on uric acid excretion in our study population, the prevalence of hyperuricemia should be higher than that of T2DM populations. However, in our study, the incidence of hyperuricemia was similar to that reported in previous studies conducted on T2DM populations in a geographically-matched rural area^26,28^. One of the reasons may be that we used newer diagnostic criteria that were higher than that used in previous studies. Another reason maybe that only a small section of our population (13.1%) had significantly impaired kidney function (eGFR < 60 mL/min/1.73 m^2^). There was a significant difference in hyperuricemia prevalence between men and women in our population, and the prevalence of hyperuricemia in males was nearly twice as high as in females, which is largely consistent with previous studies^4^.

The mechanism behind the association between the TyG index and hyperuricemia is still unclear. The TyG index is calculated from fasting plasma glucose and triglycerides, and has been proposed as a simple and reliable clinical surrogate marker for metabolic syndrome and insulin resistance^31–33^. Thus, a potential mechanism that associates the TyG index with hyperuricemia may involve insulin resistance. Studies has confirmed that glycolysis intermediates are transferred to 5-phosphoribose and phosphoric acid ribose pyrophosphate under insulin resistance, which leads to an increase in the production of serum uric acid^34^. Moreover, high levels of insulin caused by insulin resistance stimulate Na^+^-H^+^ exchange in renal tubules, increase H^+^ excretion, and increase uric acid reabsorption^35^, while the activation of the renin-angiotensin system by hyperinsulinemia decreases renal blood flow, increases urate reabsorption, and produces xanthine oxidase, which then leads to an increase in uric acid production^36^, while McCormick et al. provided robust evidence that insulin resistance has a positive causal effect on serum urate concentrations^37^. Another potential mechanism may be through blood glucose and lipid levels. On the one hand, it has been reported that hyperglycemia and hyperlipidemia decrease glyceraldehyde 3-phosphate dehydrogenase activity, which in turn increases UA synthesis^28^. On the other hand, TG can result in the renal small arteries to become stenotic or even occlusive as a result of long-term dyslipidemia, which ultimately cause urate excretion disorders. Studies have shown that the TG level is a independent and important risk factor for hyperuricemia^38^.

Our study has the following strengths. First, previous studies that reported on the associations of the TyG index and hyperuricemia were mostly cross-sectional. Our study is the first to provide a cross-sectional and longitudinal analysis to confirm an independent association between the TyG index and hyperuricemia, and included a relatively long follow-up period. Second, we were also the first study to investigate the relationship between the TyG index and hyperuricemia in the patients with DKD.

However, some limitations of this study should also be mentioned. First, although our model adjusted for many covariates, data on smoking, alcohol consumption, dietary habits, application of uric acid lowering drugs and other medications that may lower uric acid levels, duration of diabetes and socioeconomic factors, which are known to affect uric acid levels, were not available. Second, the sample size was relatively small. Third, all enrolled subjects were from a single center and only a Chinese population with DKD. Therefore, the findings of this research study may be influenced by a certain level of bias. Fourth, this is an observational study and as such cannot be used to confirm a causal relationship between the TyG index and hyperuricemia.

## Conclusion

In summary, we showed a significant independent association of the TyG index and the risk of hyperuricemia in DKD patients. Along with other known risk factors, providing simpler and more economical options that can distinguish high-risk populations to implement early stage management may help reduce the occurrence of hyperuricemia and related diseases. In addition, rigorous clinical studies will be required to further explore the relationship and mechanism involved in the association of the TyG index and hyperuricemia.

## Methods

### Study population

**Figure 4:**
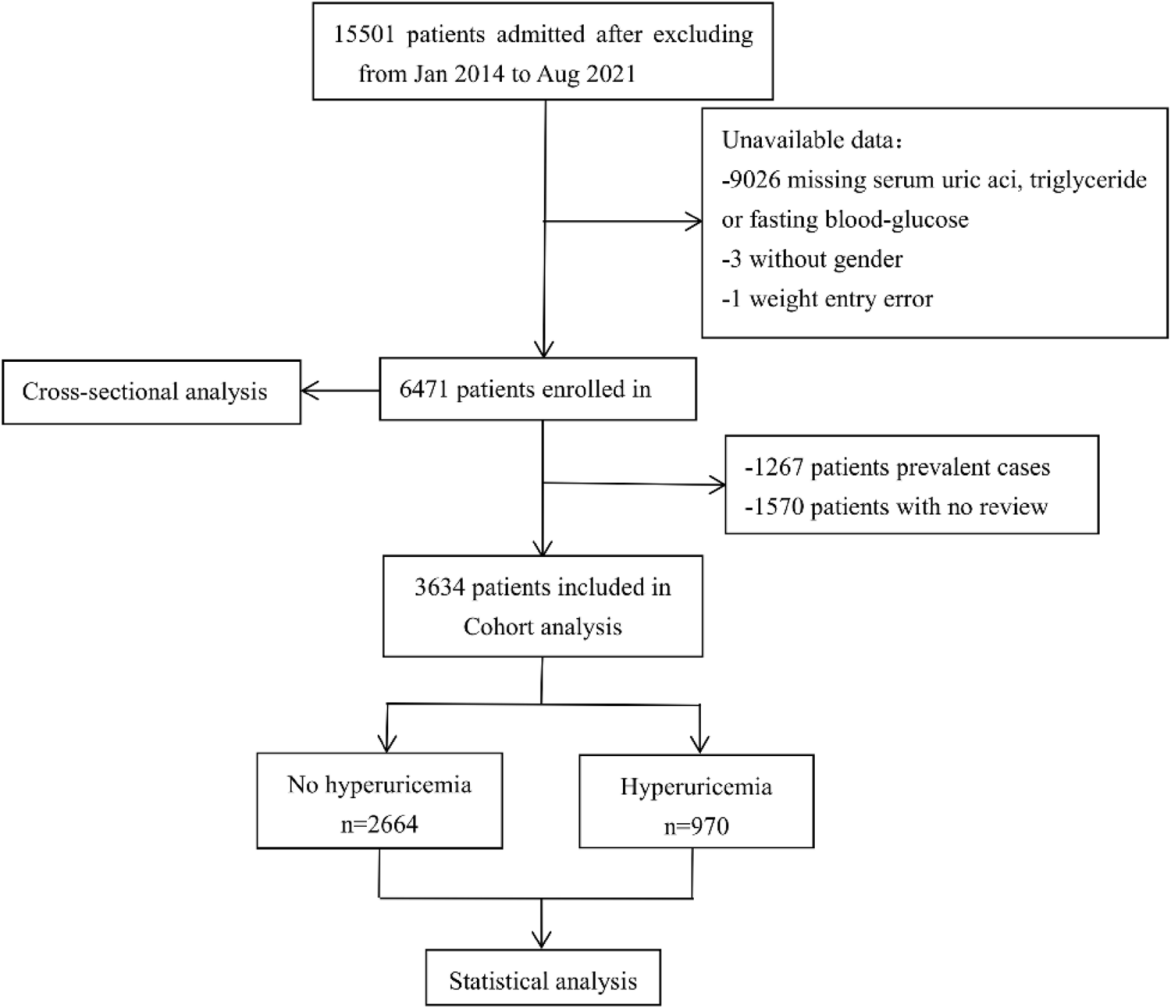
Flow chart of subject selection for this study.

All participants were selected among patients admitted to the Tianjin Medical University Chu Hsien-I Memorial Hospital from January 2014 to August 2021. DKD patients aged over 18 years old were included in our present study. DKD was defined as eGFR < 60 mL/min/ 1.73 m^2^ or continuously increasing UACR (> 30 mg/g) over a period of 3 months in type 2 diabetes mellitus (T2DM) patients. The exclusion criteria used was : (1) patients with type 1 or other special types of diabetes; (2) hepatitis B, hepatitis C, or liver function test abnormalities (alanine aminotransferase or aspartate aminotransferase value 2.5 times higher the normal level); (3) Cushing’s syndrome, hyperthyroidism, or other endocrine metabolic diseases; (4) serious diseases, such as acute cardiovascular and cerebrovascular diseases or severe infections; (5) serious complications, such as diabetic ketoacidosis and hyperosmolar hyperglycemic syndrome, or other acute metabolic disorders; (xi) pregnancy or a malignancy (xii) hypoglycemia (fasting plasma glucose (FPG) < 4 mmol/L, < 72 mg/dL). In this study, 15,501 participants were admitted after excluding. After further screening, a total of 6,471 patients were enrolled in the cross-sectional study and 3,634 patients were enrolled in the cohort study. The detailed research procedures for selecting participants was shown in Fig. 4. This study was complied with the Helsinki Declaration of 1964, as amended in 2008 and was approved by the Ethics Committee of Tianjin Medical University Chu Hsien-I Memorial Hospital.

### Data collection and laboratory assays

Height, weight, and blood pressure were obtained through medical examination. BMI is calculated by dividing the body weight in kilograms by the height in meters squared. FPG, glycosylated hemoglobin (HbA1c), lipid profiles (triacylglycerol (TG), total cholesterol (TC), high-density lipoprotein cholesterol (HDL-C), low-density lipoprotein cholesterol (LDL-C)), liver function (aspartate aminotransferase (AST), alanine aminotransferase (ALT), lactic dehydrogenase (LDH), total bilirubin (TBIL) and direct bilirubin (DBIL)) and renal function (blood urea nitrogen, and serum creatinine, serum uric acid (SUA), albumin-creatinine ratio (ACR), 24 h total urine protein(24hTP)) were evaluated through laboratory tests. All samples were processed at the Clinical Laboratory Center of Tianjin Medical University Chu Hsien-I Memorial Hospital. The estimated glomerular filtration rate (eGFR (mL/min/1.73 m^2^)) was calculated using the MDRD formula (eGFR = 186 × (Scr/88.4)^−1.154^ × (Age)^−0.203^ × (0.742 if female)). The TyG index was calculated as: TyG = ln [TG (mg/dL) × FPG (mg/dL)/2].

### Definition of covariates

Hyperuricemia was defined as serum uric acid levels of ≥ 420 umol/L (7 mg/dL) in both men and women based on the guidelines for the diagnosis and treatment of hyperuricemia and gout in China. Hypertension was defined as systolic blood pressure (SBP) of ≥ 140 mmHg or diastolic blood pressure (DBP) of ≥ 90 mmHg, measured in a resting and sitting position, or a self-reported diagnosis, or current use of an antihypertension medications.

### Statistical analysis

The baseline characteristics were showed as the mean ± standard deviation (SD) when the sample distribution was approximately normal. If the data showed a skewed distribution, the median and interquartile range (IQR) were used for continuous variables. Categorical variables were summarized as a number (percentage). Comparing baseline characteristics between different groups based on the TyG index quartiles was conducted using the analysis of variance (ANOVA) or Kruskal–Wallis test for continuous variables and was evaluated using chi-square (x^2^) tests for group variables, respectively. Multivariate logistic regression models (results presented as odds ratios (ORs) and 95% confidence interval (95% CI)) were performed to assess the relationship of TyG levels and hyperuricemia. For the time-to-event analyses, we used multivariate Cox proportional hazards regression to estimate the hazard ratios (HRs) and 95% CI of incident hyperuricemia using TyG quartiles. The cumulative risk of hyperuricemia among TyG quartiles was compared using the Kaplan–Meier survival. Furthermore, subgroup analyses were carried out to identify potential factors that could be used to evaluate the stability of our main findings. Potential interactions were tested by including interaction terms.

All data were analyzed using SPSS 24.0 software (SPSS Inc., Chicago, IL, USA), GraphPad Prism version 8.0 software (San Diego, CA, USA) and R software, version 4.2.1(http://www.R-project.org/). Two-tailed *P* value < 0.05 were considered statistical significance.

### Ethics approval and consent to participate

This study was complied with the Helsinki Declaration of 1964, as amended in 2008 and was reviewed and approved by the ethics committee of Tianjin Medical University Chu Hsien-I Memorial Hospital. Verbal informed consent was obtained from each participant and was recorded by the physician who explained the study procedures. Written informed consent was waived because the data were anonymous and observational, which was approved by the ethics committee of Tianjin Medical University Chu Hsien-I Memorial Hospital.

## Data Availability

The raw data are not available. However, the data are available from the corresponding author upon reasonable individual request.
